# Evaluation of serum phosphorus levels and their relation with the development of osteoporosis in people living with HIV/AIDS

**DOI:** 10.1590/1806-9282.20250104

**Published:** 2025-10-27

**Authors:** Thirza Damasceno Ramos Oliva, Isabella Mesquita Sfair Silva, Jeremias Estevam Lopes, Kamylla Batista Brito, Arthur Cavalcante Lopes, Rosana Maria Feio Libonati

**Affiliations:** 1 Universidade Federal do Pará, Institute of Health Sciences – Belém (PA), Brazil.; 2 Universidade Federal do Pará, Tropical Medicine Center – Belém (PA), Brazil.

**Keywords:** Hypophosphatemia, HIV, Osteoporosis

## Abstract

**OBJECTIVE:**

The aim of this study was to assess the relationship between hypophosphatemia and osteoporosis in people living with HIV on antiretroviral therapy, and to identify risk factors associated with both conditions.

**METHODS:**

This was a cross-sectional, analytical study of 96 patients at a clinic in Belém-PA. Data collection included serum phosphorus, calcium, vitamin D, parathyroid hormone, renal parameters, and bone mineral density (T-score). Student's t-test, Mann-Whitney U test, chi-square test, or Fisher's exact test was applied, as well as multiple logistic regression for hypophosphatemia and osteoporosis.

**RESULTS:**

Of the 96 patients, 24 (25%) had hypophosphatemia, with a male predominance (75%). There was a statistically significant association (p<0.05) between hypophosphatemia and longer duration of infection and use of antiretroviral therapy, as well as a higher prevalence in men. Regimens containing tenofovir disoproxil fumarate showed an increased risk of hypophosphatemia, although logistic regression did not confirm significance at the 5% level. As for osteoporosis, there was an association with female gender and age but no direct relationship with hypophosphatemia.

**CONCLUSION:**

Hypophosphatemia was significantly associated with male gender, prolonged infection, and the use of antiretroviral therapy, especially regimens with tenofovir disoproxil fumarate. There was no statistical correlation between hypophosphatemia and osteoporosis, but female gender and advanced age were risk factors for the latter. These findings highlight the importance of monitoring bone and kidney parameters in people living with HIV, especially those on long-term tenofovir disoproxil fumarate, with a view to more effective preventive and therapeutic strategies.

## INTRODUCTION

With the widespread adoption of antiretroviral therapy (ART) for people living with HIV (PLWHA), chronic and degenerative conditions have become increasingly prevalent. Among these, hypophosphatemia and osteoporosis substantially impair quality of life, as chronic inflammation, ART side effects, and metabolic disturbances exacerbate bone-related complications in this population^1^. Osteoporotic fractures, particularly of the hip and spine, are linked to higher morbidity and mortality, underscoring the importance of early detection and management.

Hypophosphatemia, defined by abnormally low serum phosphate levels, disrupts energy metabolism, bone mineralization, and cellular signaling. Osteoporosis, marked by reduced bone mass and microarchitectural deterioration, heightens fracture risk. In HIV-infected individuals, metabolic derangements further promote phosphate depletion and bone demineralization. Although ART remains essential for viral suppression, certain regimens have been associated with decreased bone mineral density and altered calcium–phosphate homeostasis^2^.

Given the functional and clinical burden of hypophosphatemia and its association with osteoporosis and fractures in PLWHA, this study evaluates their interrelationship and identifies risk factors for both conditions in patients receiving ART. By elucidating the mechanisms underlying bone health impairment, our findings aim to inform targeted screening and intervention strategies to mitigate adverse outcomes and enhance long-term well-being in this vulnerable group.

## METHODS

This cross-sectional analytical study was conducted at the Endocrinology Clinic of the Tropical Medicine Center at the Federal University of Pará, using field and documentary research methods. We enrolled 96 HIV-positive adults on ART, selected by convenience sampling; based on a 23.15% osteoporosis prevalence^3^, a 95% confidence interval, and 5% maximum error, a minimum sample of 77 was required. The study adhered to Strengthening the Reporting of Observational Studies in Epidemiology (STROBE) guidelines^4^.

Inclusion criteria comprised age ≥18 years, confirmed HIV infection, and ongoing ART; prior renal failure or hemodialysis led to exclusion. Data sources included medical records and outpatient assessments. We measured serum phosphorus, calcium, 25-hydroxyvitamin D, parathormone, creatinine, urea, uric acid, proteinuria, and microalbumin. Bone mineral density (T-score) was collected and analyzed. Estimated glomerular filtration rate (eGFR) (mL/min/1.73 m^2^) was calculated via the CKD-EPI equation and categorized as G1 (≥90), G2 (60–89), G3a (45–59), G3b (30–44), G4 (15–29), or G5 (<15). Exposure variables were age, sex, duration of HIV infection, ART regimen, and ART duration; outcomes were hypophosphatemia (<2.5 mg/dL) and osteoporosis.

Statistical analyses were performed in Excel 2017^®^, BioEstat 5.3, Epi Info 7.0, and Python 3.13.0. Bivariate comparisons were performed using Student's t-test, Mann-Whitney U test, chi-square test, or Fisher's exact test, as appropriate. Variables with p<0.05 entered multiple logistic regression models for hypophosphatemia and osteoporosis. To assess non-significant findings, post hoc power analyses were conducted for all primary comparisons using a chi-square framework, with α=0.05 and target power ≥80%. Prospective power calculations are essential in observational designs to avoid Type II errors and ensure adequate sample sizes for detecting clinically meaningful associations^5^.

## RESULTS

The study included 96 patients, of whom 53 (55.20%) were male and 43 (44.80%) were female. Ethnically, 33 (34.38%) patients were White, 21 (21.88%) Black, 41 (42.70%) Brown, and 1 (1.04%) Asian. Additionally, 24 (25.00%) patients presented with hypophosphatemia, while 72 (75.00%) had normophosphatemia. The epidemiological data are shown in Table 1:

**Table 1:** Mean±standard deviation of age, absolute and relative frequencies of genders, and median interquartile deviations of years of infection and years of antiretroviral therapy use, followed by the respective p-values in people living with HIV with and without hypophosphatemia (Thirza Damasceno Ramos Oliva, 2022).

Variables	Hypophosphatemia (<2.5 mg/dL)	Normophosphatemia (2.5–5.6 mg/dL)	p-value
Age	59.95±9.28	54.91±11.71	0.058*
Gender	18 (75%)	35 (48.61%)	**0.024** **
	6 (25%)	37 (51.39%)	
Time since diagnosis of infection (years)	22.00 (9.25)	12.50±13.00	<0.0013***
Time on ART (years)	22.00 (9.50)	12.00±13.25	<0.0011***

* T-student.

** Chi-square test.

*** Mann-Whitney U test; ART: antiretroviral therapy. Statistically significant values (p<0.05) are denoted in bold.

In the analysis of laboratory variables, no statistically significant differences were observed between normophosphatemic and hypophosphatemic patients for creatinine, urea, total calcium, 25-hydroxyvitamin D, parathormone, uric acid, proteinuria, or microalbumin levels (Mann-Whitney U test, all p>0.05). To evaluate renal function, eGFR stages were compared: among normophosphatemic patients, 9 (12.5%) were in stage 1, 34 (47.22%) in stage 2, 20 (27.78%) in stage 3A, 7 (9.72%) in stage 3B and 2 (2.78%) in stage 4, with none in stage 5; in the hypophosphatemic group, 1 (4.16%) was in stage 1, 11 (45.84%) in stage 2, 7 (29.17%) in stage 3A, and 5 (20.83%) in stage 3B, with no patients in stage 4 or 5, demonstrating no significant association between phosphate status and eGFR (Mann-Whitney U test, p=0.372).

CD4^+^ T-lymphocyte counts had a median of 727,50 cells/μL and an interquartile range (IQR) (371,25 cells/μL) in hypophosphatemic patients, in comparison to 610,50 cells/μL (IQR: 327,00 cells/μL) in normophosphatemic patients; this difference did not reach statistical significance (Mann-Whitney U test, p=0.091).

Regarding bone health, five of 24 hypophosphatemic patients (20.83%) had osteoporosis compared to 12 of 72 normophosphatemic patients (16.67%; p=0.758); reduced bone mineral density (osteoporosis or osteopenia) was present in 62.50% of both groups (p=1.000). A significant relationship emerged between tenofovir disoproxil fumarate (TDF) exposure and hypophosphatemia: the median duration of TDF use was 22.00±11.00 years in the hypophosphatemic group, compared with 12.00±15.25 years in the normophosphatemic group (Mann-Whitney U test, p=0.002). Phosphorus levels in TDF users ranged from 2.2 to 2.4 mg/dL, whereas those in non-users ranged from 2.6 to 3.8 mg/dL, which was also statistically significant (Mann-Whitney U test, p=0.0278).

In the analysis of bone mineral density, there was a significant association between age and gender and the presence of osteoporosis, but there was no statistical relationship between the use of TDF and the development of osteoporosis (Table 2).

**Table 2:** Median±interquartile range for age, absolute and relative frequencies of gender, and use of tenofovir disoproxil fumarate for patients with and without osteoporosis, followed by the respective p-values in people living with HIV (Thirza Damasceno Ramos Oliva, 2022).

Variables		No (osteoporosis)	Yes (osteoporosis)	p-value
Age		54±14.5	61±13	0.001*
Gender				
	Male	49 (62.02%)	4 (23.52%)	0.006**
	Female	30 (37.98%)	13 (76.49%)	
Use of TDF				
	Yes	60 (75.94%)	11 (64.70%)	0.3379***
	No	19 (24.06%)	6 (35.3%)	

TDF: tenofovir disoproxil fumarate.

* Mann-Whitney U test.

** Fisher's exact test.

*** Chi-square test.

In several primary analyses, we did not observe statistically significant associations. To clarify whether this may have been due to limited sample size, post hoc power analyses were conducted. The power to detect differences in osteoporosis prevalence between hypophosphatemic and normophosphatemic patients was 11.9%. For the prevalence of reduced bone mineral density (osteopenia or osteoporosis), where proportions were identical in both groups, the power was 5%. The power for the comparison of impaired glomerular filtration rate (GFR<60 mL/min/1.73 m^2^) between the groups was 39.4%. For the association between TDF use and osteoporosis, the power was 18.9%.

Regarding the comparison of CD4+ T-lymphocyte counts (median: 727.5 [IQR: 371.25] and 610.5 [IQR: 327], for the respective groups, p=0.091), the estimated effect size was 0.47, and the post hoc power was 50.6%. All of these values fall well below the generally accepted threshold of 80% for adequate power, indicating a considerable risk of type II error (false negatives) in these analyses.

To analyze the outcomes of hypophosphatemia and osteoporosis, two multiple logistic regressions were carried out, each involving variables that showed significance in the bivariate analysis for the respective outcome. The variable "years since diagnosis" was excluded from the regression due to collinearity with the variable "years since treatment" (variance inflation factor [VIF]=5.82). Male sex was identified as a risk factor for the development of hypophosphatemia. In contrast, female sex was considered a risk factor for the development of osteoporosis. The data are organized in Table 3.

**Table 3:** Multiple logistic regression analyses of factors associated with hypophosphatemia and osteoporosis in people living with HIV (Thirza Damasceno Ramos Oliva, 2022).

Variables	Coefficient	Standard error	Z	p-value	Odds ratio	95%CI
Hypophosphatemia	-3.8885	0.9442	–	–	–	–
Gender Male=(1); Female=(0)	0.9961	0.5550	1.7948	0.0727	2.7078	0.91–8.04
Years of treatment	0.0899	0.0332	2.7070	**0.0068**	1.0941	1.03–1.17
TDF Yes=1; No=0	0.9511	0.7123	1.3351	0.1818	2.5885	0.64–10.46
**Variables**	**Coefficient**	**Standard error**	**Z**	**p-value**	**Odds ratio**	**95%CI**
Osteoporosis	-11.2134	2.8204	–	–	–	–
Gender Female=1; Male=0	2.6177	0.7833	3.3417	**0.0008**	13.7035	2.95–63.62
Age	0.1371	0.0409	3.3556	**0.0008**	1.1469	1.06–1.24

CI: confidence interval; TDF: tenofovir disoproxil fumarate.

## DISCUSSION

Our study uncovered an association between hypophosphatemia and TDF use, alongside male gender, diagnosis duration, and ART length. Thus, we infer that hypophosphatemia is linked to tenofovir and raises the risk of reduced bone mineral density. Although ART modifies HIV progression and extends survival, the widespread use of tenofovir and related agents has escalated metabolic irregularities, posing new clinical challenges^6^.

Regarding the duration of HIV infection and ART, normophosphatemic individuals in our sample had median values of 12.5 years for infection and 12 years for ART, while hypophosphatemic participants had medians of 22 years for both. Thus, extended time living with HIV and receiving ART correlates with hypophosphatemia, consistent with findings from Swiss cohort study^7^.

Among the normophosphatemic participants, the average GFR was 64.95±25.52 standard deviation, while the hypophosphatemic individuals had a mean score of 60.40±24.57 standard deviation. A cohort study^8^ of 3,714 people showed different results, with 66.1% in G1, 29.8% in G2, 0.6% in G3B, and only 0.4% in G4 or G5. Furthermore, among individuals not receiving ART, GFR <90 mL/min/1.73 m^2^ occurs in 25% of HIV-infected patients^9^.

In this investigation, 12 (16.7%) normophosphatemic participants had osteoporosis and 45 (62.5%) presented with reduced bone mineral density (osteoporosis or osteopenia), whereas 27 (37.5%) showed normal results. In a Brazilian study, 54% had reduced bone density^10^. Among Japanese men with HIV-1, 54% had osteopenia and 12% had osteoporosis^11^, findings that are comparable to those in our study.

Acute or chronic phosphorus deficiency impairs bone tissue, hindering matrix formation, osteoid maturation, and mineralization while promoting osteoclastic activity^12^. Nonetheless, our findings revealed no significant link between low serum phosphorus and reduced bone mineral density or osteoporosis. This may be explained by compensatory mechanisms, particularly in the kidney, where phosphate reabsorption is regulated by adjusting the apical abundance of Na^+^/Pi cotransporters (mainly NaPi-IIa and NaPi-IIc)^13^.

Although TDF has been tied to higher osteoporosis risk and fractures^14^, we found no statistical significance. A Japanese survey of HIV-infected individuals reported a low overall fracture incidence, but women experienced a fracture rate three times higher, partly because they were about 20 years older at onset^15^. The chronic inflammatory state in HIV—marked by elevated tumor necrosis factor-α (TNF-α) and interleukin-6 (IL-6)—enhances osteoclastogenesis and bone resorption. Hypoestrogenism in postmenopausal women further amplifies this imbalance in bone remodeling^16^. These results match global patterns linking advanced age and female gender to osteoporosis^17^.

Our regression analysis showed that each additional ART year increased hypophosphatemia odds by 9.4%. Male gender approached but did not reach significance at 5%. Tenofovir use was not significantly associated. For osteoporosis, female gender and age were independent risk factors, with the odds increasing by 14.7% per year. These findings inform targeted prevention and treatment strategies to enhance clinical outcomes and patient care in HIV-infected patients.

This study has some limitations that should be considered. Our sample of 96 patients, with only 24 showing hypophosphatemia, may limit extrapolation. We also did not control for confounders such as dietary phosphorus intake, physical activity levels, body mass index (BMI), smoking status, and alcohol consumption, although these factors may influence the study^18^. Our use of convenience sampling and the relatively homogeneous sample may introduce selection bias and limit the generalizability of these results. Lastly, the sample's limited geographic and ethnic range restricts the generalizability of these results.

## CONCLUSION

Male gender, longer infection duration, and extended ART, particularly regimens containing TDF, were significantly associated with hypophosphatemia. No direct link with osteoporosis was observed, although female gender and older age emerged as risk factors for reduced bone density. These findings underscore the need for regular monitoring of bone and renal parameters in PLWHA on long-term TDF and highlight the importance of future longitudinal studies with larger, more heterogeneous samples to validate and expand upon these results.

## Data Availability

The datasets generated and/or analyzed during the current study are available from the corresponding author upon reasonable request.
